# Concurrent anxiety in patients with major depression and cerebral serotonin 4 receptor binding. A NeuroPharm-1 study

**DOI:** 10.1038/s41398-022-02034-5

**Published:** 2022-07-11

**Authors:** Kristin Köhler-Forsberg, Brice Ozenne, Søren V. Larsen, Asbjørn S. Poulsen, Elizabeth B. Landman, Vibeke H. Dam, Cheng-Teng Ip, Anders Jørgensen, Claus Svarer, Gitte M. Knudsen, Vibe G. Frokjaer, Martin B. Jørgensen

**Affiliations:** 1 Neurobiology Research Unit, Copenhagen University Hospital Rigshospitalet, Copenhagen, Denmark; 2 Institute of Clinical Medicine, University of Copenhagen, Copenhagen, Denmark; 3 Psychiatric Centre Copenhagen, Rigshospitalet, Copenhagen, Denmark; 4 Department of Public Health, Section of Biostatistics, University of Copenhagen, Copenhagen, Denmark; 5 Department of Clinical Pharmacology, H. Lundbeck A/S, Valby, Denmark

**Keywords:** Predictive markers, Molecular neuroscience

## Abstract

Concurrent anxiety is frequent in major depressive disorder and a shared pathophysiological mechanism between anxiety and other depressive symptoms is plausible. The serotonin 4 receptor (5-HT_4_R) has been implicated in both depression and anxiety. This is the first study to investigate the association between the cerebral 5-HT_4_R binding and anxiety in patients with depression before and after antidepressant treatment and the association to treatment response. Ninety-one drug-free patients with depression were positron emission tomography scanned with the 5-HT_4_R ligand [^11^C]-SB207145. Depression severity and concurrent anxiety was measured at baseline and throughout 8 weeks of antidepressant treatment. Anxiety measures included four domains: anxiety/somatization factor score; Generalized Anxiety Disorder 10-items (GAD-10) score; anxiety/somatization factor score ≥7 (anxious depression) and syndromal anxious depression. Forty patients were rescanned at week 8. At baseline, we found a negative association between global 5-HT_4_R binding and both GAD-10 score (*p* < 0.01) and anxiety/somatization factor score (*p* = 0.06). Further, remitters had a higher baseline anxiety/somatization factor score compared with non-responders (*p* = 0.04). At rescan, patients with syndromal anxious depression had a greater change in binding relative to patients with non-syndromal depression (*p* = 0.04). Concurrent anxiety in patients with depression measured by GAD-10 score and anxiety/somatization factor score is negatively associated with cerebral 5-HT_4_R binding. A lower binding may represent a subtype with reduced natural resilience against anxiety in a depressed state, and concurrent anxiety may influence the effect on the 5-HT_4_R from serotonergic antidepressants. The 5-HT_4_R is a promising neuroreceptor for further understanding the underpinnings of concurrent anxiety in patients with depression.

## Introduction

While anxiety is not a formal ICD/DSM symptom of depression, it is commonly observed in depressed patients and has prognostic importance [1]. Hence, it is weighted high and included in standard rating scales for depression, e.g. the Hamilton Depression Rating Scale (HAMD) [2]. While anxiety disorders have a prevalence of 284 million people worldwide [3], comorbidity of anxiety disorders or significant levels of anxious symptoms are present in up to 85% of patients with Major Depressive Disorder (MDD) [1, 4–7]. The presence of concurrent anxiety in depression has been associated with more and worse side effects to antidepressant medication, longer time to remission, greater severity and poorer treatment outcome [8, 9]. The underlying mechanisms are not fully understood, but serotonin (5-HT) is assumed to play a key role and serotonergic-acting drugs are first-line pharmacotherapeutic options for treatment of both depression and anxiety disorders. Previous positron emission tomography (PET) studies of the serotonin system in patients with concurrent anxious depression are scarce and have mainly focused on the serotonin transporter (SERT) and the 5-HT_1A_ receptor [10, 11]. One study found that low SERT binding in thalamus was correlated with higher anxiety symptoms in a small sample of ten unmedicated patients with MDD [11] which was later replicated (*n* = 10) [12]. Another group confirmed a negative correlation between somatic anxiety and SERT binding in the thalamus, midbrain and amygdala, while psychic anxiety was positively correlated with midbrain SERT binding [10].

There is growing evidence for involvement of the 5-HT 4 receptor (5-HT_4_R) in anxiety and MDD, which is a G_s_-protein coupled receptor abundant in neostriatum, the limbic region and prefrontal cortex [13–15]. Studies have demonstrated that 5-HT_4_R modulation is associated with anxiolytic-like behaviour in rodents, e.g., 5-HT_4_R knockout mice show attenuated novelty seeking behaviour [16] and both acute and (sub)chronic 5-HT_4_R stimulation elicits anxiolytic and antidepressant-like behaviour [17, 18]. Further, chronic 5-HT_4_R agonism treatment was found to prevent depressive- and anxiety-like behaviour [19]. Due to the observed antidepressant and anxiolytic properties, 5-HT_4_R agonists have been proposed as a new/add-on pharmacological target in anxiety and MDD [19, 20]. To our knowledge, only one study has investigated the effect of 5-HT_4_R agents in humans; a single oral administration of the partial 5-HT_4_R agonist prucalopride exerted pro-cognitive effects in healthy individuals, but had little effect on emotional processing related to an antidepressant profile [21]. Interestingly, preclinical and clinical evidence suggest that the 5-HT_4_R may serve as an inverse biomarker of the cerebral serotonin tonus [22–24], which makes it a relevant candidate when studying conditions with a presumed serotonergic involvement. The implications of 5-HT_4_R in MDD have been recognized [25] and recently, we showed that antidepressant-free patients with MDD had lower 5-HT_4_R binding compared with healthy controls, especially those responding well to escitalopram [26].

We here aim, for the first time, to investigate the association between 5-HT_4_R PET binding and concurrent anxious symptomatology in patients with MDD. We also investigate the association between concurrent anxious depression and antidepressant treatment response. Based on the previous findings of increased anxiety- and depressive-like behaviour in 5-HT_4_R knock-out mice and the anxiolytic effects from 5-HT_4_R stimulation in rodents [18, 27], we hypothesized that higher anxiety symptoms at baseline would be associated with lower 5-HT_4_R binding in patients with MDD. We also hypothesized that having concurrent anxiety at baseline would be associated with worse treatment response, that a change in 5-HT_4_R binding after 8 weeks of serotonergic antidepressant treatment would be associated with higher baseline anxiety, and that a change in binding would be associated with a change in anxiety score.

## Methods and materials

The present paper is part of the NeuroPharm-1 study; a first-time, non-randomized, open-label clinical trial conducted between August 2016 to April 2019. Recruitment took place at a referral centre within the Mental Health System or at collaborating primary care centres in the capital region of Denmark. All participants provided written informed consent before participation. The study was pre-registered at clinicaltrials.gov (ID: NCT02869035) and complied with regulations from the Committees on Health Research Ethics in the Capital Region of Denmark (ID: H-15017713), the Danish Medicines Agency (ID: NeuroPharm-NP1) and the Danish Data Protection Agency (ID: 04711/RH-2016-163). The study is detailed in the trial protocol [28]. The participants were also included in a study of the association between 5-HT_4_R and MDD as primary outcome [26], whereas the anxiety measures presented in this study served as exploratory outcomes.

### Participants

One hundred patients were included to reach statistical power of 0.8, for detection of a 7% difference in BP_ND_ between remitters and non-responders in the primary study [26, 28]. The expected drop-out rate was 20%. Ninety-one out of the recruited 100 planned patients completed a PET scan at baseline. All patients had unipolar, moderate to severe MDD according to DSM-IV criteria [29]. Depression severity was assessed with the HAMD-17 items (HAMD_17_) where all patients had a baseline score >17. Patients were 18–65 years and medically untreated for their depression for at least 2 months prior to inclusion. The duration of the current depressive episode did not exceed 2 years. Exclusion criteria were psychotic manifestations; acute suicidal ideations; alcohol abuse/substance use disorder; another primary axis I psychiatric diagnosis; previous non-response to an selective serotonin reuptake inhibitor (SSRI); central acting drugs that could not be washed out prior to scanning; pregnancy/breast feeding; post-concussion syndrome and severe somatic comorbidity.

### Baseline assessments

Patients filled out the questionnaire of Generalized Anxiety Disorder 10 items (GAD-10) [30] and were interviewed with the Mini International Neuropsychiatric Interview (MINI) [31]. Baseline assessments further included medical history; somatic examination; routine bloodwork; urine pregnancy and toxicology tests; blood test for genetic variation of the serotonin transporter-linked polymorphic region (5-HTTLPR); and magnetic resonance imaging (MRI)- and PET scans.

### Treatment programme

After baseline assessments, patients started antidepressant treatment with escitalopram at flexible doses between 10–20 mg daily, individually adjusted depending on side effects and clinical response. Face to face clinical follow-up sessions were conducted after 1, 2, 4, 8 and 12 weeks. Additional visits were accepted if clinically justified. Treatment response was monitored at each follow-up session using the HAMD_17_ and the subscale of 6 items (HAMD_6_), collected by a trained physician or study assistant. Co-ratings for HAMD calibration was held monthly. Drug compliance was assessed by pill-count at each visit and trough serum blood samples at week 8. Short acting sleeping pills and anxiolytics (benzodiazepines) were accepted to reduce initial side effects, but not 72 h prior to PET-scans. If patients had excessive side effects or insufficient response to escitalopram after 4 weeks, they were offered to switch to duloxetine. Duloxetine is a standard second line antidepressant treatment and has negligible affinity to the 5-HT_4_R [32]. After 8 weeks, a subgroup of patients (*n* = 43) was rescanned.

### Characterization of anxious depression

Concurrent anxious depression was measured by (A) the HAMD_17_ anxiety/somatization factor score (here referred to as “factor score”) [8]. The factor score was derived from a HAMD_17_ factor analysis by Cleary and Guy [33] and included six items: item 10 (psychic anxiety), 11 (somatic anxiety), 12 (gastrointestinal somatic symptoms), 13 (general somatic symptoms), 15 (hypochondriasis) and 17 (insight). We also used (B) the GAD-10 questionnaire (“GAD-10 score”) which is an inventory for generalized anxiety distress symptoms [30]. For the categorical outcomes, we used (C), a previously established definition of high levels of anxiety in depressed patients, where “anxious depression” was defined as a HAMD_17_ somatization/anxiety factor score ≥7 and “non-anxious depression” as a score <7 [8]. Lastly, (D) “syndromal anxious depression” was defined as having at least one concurrent anxiety diagnosis identified by the M.I.N.I. interview [34, 35], including panic disorder, generalized/not generalized social phobia, agoraphobia and generalized anxiety (associated with the depressed episode). Correspondingly, patients with “non-syndromal” anxious depression had no additional anxiety diagnosis verified by the M.I.N.I. interview. GAD-10 was a pre-registered outcome, whereas the other anxiety measures were exploratory and chosen because they are previous established outcomes [8, 34, 35].

### Definitions of clinical treatment outcome

The clinical treatment outcome was based on changes in HAMD_6_ from baseline. HAMD_6_ was chosen because of its superior properties in monitoring treatment response compared to HAMD_17_ [36]. The primary treatment outcome was composed of two response categories at week 8: patients were categorized as remitters if they had an early response of ≥50% reduction in HAMD_6_ at week 4 and a HAMD_6_ < 5 at week 8; non-responders had an early non-response of <25% reduction in HAMD_6_ at week 4 and <50% HAMD_6_ reduction at week 8. Additionally, patients in between these categories were referred to as intermediate responders at week 8. This construct was applied to capture early (week 4) and sustained (week 8) treatment response. The secondary treatment outcome measure was a continuous outcome defined as percentage change in HAMD_6_ at week 8 relative to baseline (r∆HAMD_6_).

### Brain regions of interest

Regions of interest (ROI) were set to neocortex, neostriatum and hippocampus. These regions display high density of 5-HT_4_R [13], are thought to be involved in MDD and were chosen to align with several studies of the serotonin system in MDD [22, 28, 37, 38].

### PET acquisition and kinetic modelling

The PET acquisition and quantification has been described in detail elsewhere [28]. Briefly, all patients were scanned with a High-resolution Research Tomography (HRRT) PET scanner (CTI/Siemens, Knoxville, TN, USA) for 120 min after an intravenous 20 s bolus injection of [^11^C]-SB207145, and a 6 min transmission scan. The PET data was reconstructed into 38 time frames (6 × 5 s, 10 × 15 s, 4 × 30 s, 5 × 2 min, 5 × 5 min, and 8 × 10 min) and motion correction was performed with Air.5.2.5 [39], aligning each PET frame to the first 5 min frame (frame 26). All patients were MR-scanned using a Siemens 3-Tesla Prisma scanner, and T1 weighted MRI images were co-registered with the PET images to acquire anatomical information using SPM8. ROIs were automatically extracted using the pvelab software package [40] and delineated on each subject’s MRI. Correct co-registration of PET and MR images and ROI placement were visually quality checked in three planes by a trained investigator. Mean time activity curves for hemisphere weighted grey matter volumes were used in the kinetic modelling and the simplified reference tissue model with cerebellum (excluding vermis) as reference region [41, 42] yielded non-displaceable binding potential (BP_ND_) as outcome measure.

### Statistical analysis

#### Association between baseline 5-HT_4_R binding and concurrent anxiety

A latent variable model (LVM) [43] was used to jointly model the 5-HT_4_R BP_ND_ across brain regions and concurrent anxious depression at baseline. A separate LVM was used for each of the four anxiety measures. It included a single latent variable representing the three brain region’s 5-HT_4_R binding with neocortex as a reference (“global BP_ND_”) and its relation to anxiety measures. Score tests were used to detect model misspecifications in the covariance structure and additional parameters were included until no misspecification could be detected.

5-HT_4_R BP_ND_ values in the LVM were log-transformed. In the primary baseline analyses, we tested for an association between global BP_ND_ and (A) factor score and (B) GAD-10 score. In a secondary analysis, we tested for a difference in global BP_ND_ between (a) anxious vs. non-anxious depression and (b) syndromal vs. non-syndromal anxious depression. If an effect was found, the association between 5-HT_4_R BP_ND_ in each brain region and the anxiety-measure was tested.

#### Association between baseline anxiety and treatment outcome (longitudinal analyses)

In the primary longitudinal analyses, we used a univariate linear regression model to test for an association between either (I) baseline factor score or (II) baseline GAD-10 score and categorical treatment outcome at week 8. Secondary, we tested for a difference in r∆HAMD_6_ in (III) anxious vs. non-anxious depression and (IV) syndromal vs. non-syndromal anxious depression at baseline.

#### Anxiety as a prognostic biomarker of treatment response (prediction analyses)

A receiver operating characteristic (ROC) curve was used to visualize the ability of baseline anxiety to discriminate between treatment remitters and non-responders from “all other” patients (i.e., intermediate responders combined with either non-responders or remitters respectively). The predictive ability of baseline anxiety was then summarized using area under the curve (AUC). On top of the clinical covariates, we tested the added value of anxiety by comparing AUCs between the predicted probabilities of a logistic model with and without anxiety. No cross-validation was used to correct the optimism in the estimated AUC because the optimism was expected to be small when assessing the anxiety effect (only 1 more degree of freedom when including this variable).

#### Association between change in 5-HT_4_R binding and concurrent baseline anxiety

In the rescan analyses, we used an LVM where we primarily tested if a change in BP_ND_ was associated with concurrent baseline anxiety; (1) syndromal vs. non-syndromal anxious depression and (2) anxious vs. non-anxious depression. Secondary, we tested if a change in global BP_ND_ was associated with a change in (3) factor score or (4) GAD-10 score from baseline to week 8.

An LVM has the advantage of no need for adjustment of multiple comparison at a global level since all regions are tested jointly. Regionally, *p*-values and 95% confidence intervals (CI) were adjusted for multiple comparisons using the single-step Dunnett procedure [44] denoted “*p*.adj”, unless otherwise specified. All tests were two-sided. Baseline analyses were adjusted for sex, age, injected tracer (mass/kg), 5-HTTLPR genotype status (L_A_L_A_ or non-L_A_L_A_) [37, 45–47] and HAMD_17/6_ item 1 + 2 (depressed mood and feelings of guilt, i.e. non-anxious core depression scores). For the longitudinal and prediction analyses, we corrected for age, sex, HAMD_17_ item 1 + 2 and for use of benzodiazepines. The rescan analyses were corrected for the difference in injected tracer (mass/kg) and for the difference in HAMD_17_ item 1 + 2 between baseline and rescan. Missing data from the baseline PET-scan was considered missing completely at random. The longitudinal analyses were performed as complete case analyses and patients with non-compliance at week 8 were not included in these analyses. Violations of normality assumptions were inspected with Q–Q plots and all BP_ND_ values were log-transformed and at last back-transformed.

## Results

Demographics and patient-characteristics are shown in Table 1. One hundred patients entered the study, nine of these did not complete the baseline PET-scan and two patients failed to fill in the baseline GAD-10 questionnaire. Seventy-eight patients (13 non-responders, 43 intermediate responders and 22 remitters) were included in the longitudinal analyses. One non-responder did not return the baseline GAD-10 questionnaire; 9 patients were excluded for various reasons (spontaneous remission (*n* = 1), acute psychosis (*n* = 1), intolerable side effects (*n* = 1), self-reported non-compliance (*n* = 1), withdrawal of consent (*n* = 5)), and four patients were excluded due to undetectable serum drug levels at week 8. Six patients were switched to duloxetine before week 8. Due to side-effects, one patient (remitter) maintained a therapeutic dose of 5 mg escitalopram and had a serum escitalopram level within one standard deviation from the mean of the group at week 8. Six patients received short-term benzodiazepines before week 8. Forty-three patients were allocated to the rescan-programme, three of those were excluded from the analyses because of non-compliance (*n* = 2) and PET-scan failure (*n* = 1). See Fig. S1 (CONSORT-diagram) for an overview. No serious adverse event occurred.

**Table 1:** Demographics and clinical profile of patients at baseline.

		*N*	%
Sex	Female	65	71.4
	Male	26	28.6
5-HTTLPR genotype	L_A_LA	26	28.6
	Non-LALA	65	71.4
Syndromal anxious depression	Yes	42	46.2
	No	49	53.8
Anxious depression	Yes	75	82.4
	No	16	17.6
Severity of MDD	Severe	32	35
	Moderate	59	65
	**Mean (SD)**	**n**	**Range**
Age (years)	27.1 (8.2)	91	18.3–57.3
Factor score	7.9 (2.0)	91	3–15
GAD-10 score	24.0 (9.3)	89	7–47
HAMD_17_ score	22.9 (3.4)	91	18–31
HAMD_6_ score	12.3 (1.6)	91	7–17
Years of education	11.6 (1.1)	76	5–12
Body mass index (kg/m^2^)	24.5 (5.6)	91	17–45
Injected dose (MBq)	577.4 (56)	91	263–615
Injected mass/kg (µg/kg)	0.013 (0.015)	91	0.004–0.082
Cerebellum, area under curve (kBq/ml)	10.3 (2.6)	91	3.9–17.8

5-HTTLPR; serotonin transporter-linked *polymorphic* region. MDD; Major depressive disorder. Factor score; total score from HAMD_17_-items 10- 13, 15 and 17. GAD-10; Generalized anxiety disorder 10 items. HAMD_17+6_; Hamilton depression rating scale 17 and 6 items.

### Baseline outcomes

**Fig. 1:**
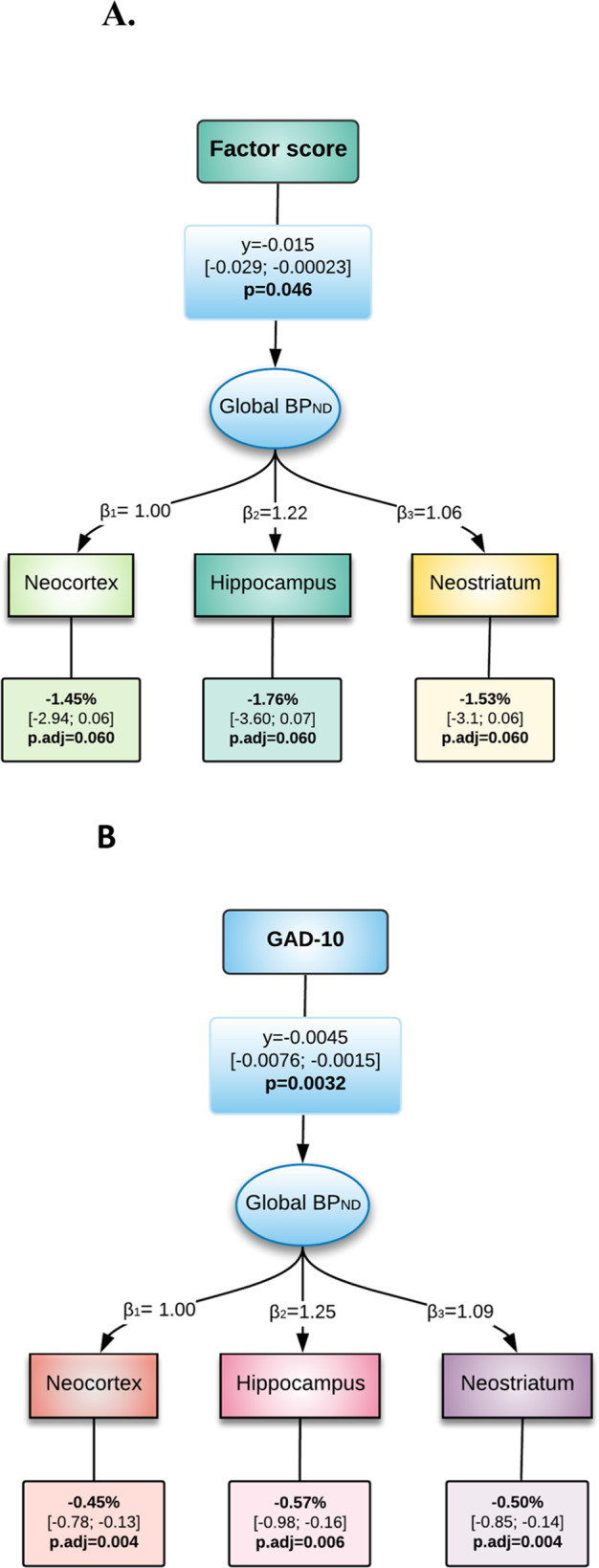
LVM of Baseline Scores. **A** Latent variable model of log-transformed baseline 5-HT_4_R BP_ND_ and factor score. **B** Latent variable model of log-transformed baseline 5-HT_4_R BP_ND_ and GAD-10. Factor score: Anxiety/somatization factor score. GAD-10: Generalized Anxiety Disorder 10 items. The lower boxes contain the percent change in regional binding for each unit increase in factor-score and GAD-10 score. β and γ correspond to the effects on the log-transformed binding.

**Fig. 2:**
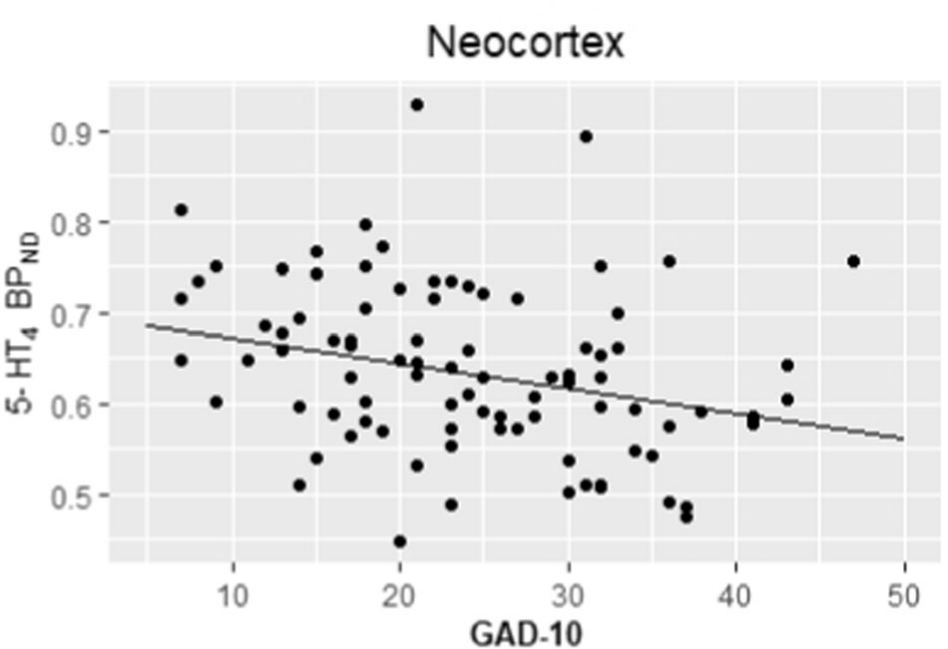
Partial residual plot showing the association between baseline 5-HT_4_R BP_ND_ in neocortex and GAD-10 (adjusted for covariates). Adjustment is performed using a linear regression on the log-transformed binding, subtracting the covariate effects compared to a female subject, age 25, non-LALA, injected mass/kg of 0.0085 and a HAMD item 1 + 2 score of 5 as reference.

The LVM identified a negative association between the global 5-HT_4_R BP_ND_ and anxiety factor score (*p* = 0.046). At a regional level, there was a negative association between baseline BP_ND_ and factor score, spanning between 1.45% to 1.76% lower binding per anxiety factor score in neocortex, hippocampus and neostriatum (*p*.adj = 0.06) (Fig. 1A). A significant negative association was found between baseline global BP_ND_ and GAD-10 score (*p* < 0.01) (Fig. 1B). Regionally, there was between 0.45% to 0.57% lower 5-HT_4_R binding per GAD-10 item score across all tested regions (*p*.adj < 0.01) (Fig. 2 shows neocortex as a representative example). We found no evidence for a difference in global BP_ND_ in patients depending on anxious depression (*n* = 75, *p* = 0.52) or syndromal anxious depression (*n* = 42, *p* = 0.11).

### Longitudinal outcomes

**Fig. 3:**
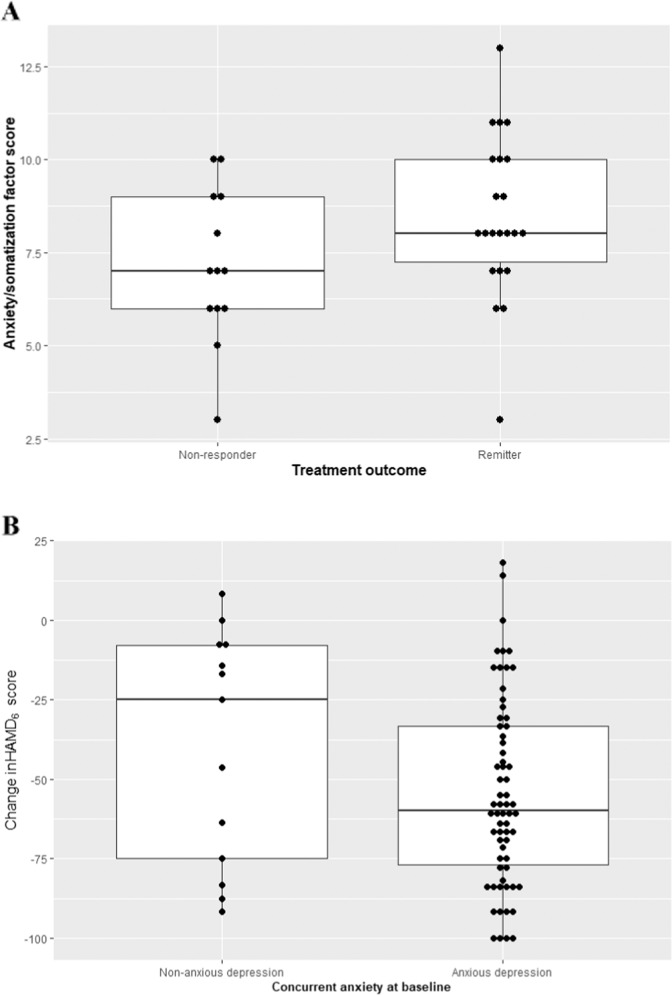
Longitudinal outcomes in anxious and remitters. **A** Baseline factor score in MDD non-responders and remitters as defined after 8 weeks of treatment. **B** Change in HAMD score from baseline to week 8 in patients with concurrent anxious vs. non-anxious depression at baseline.

The baseline factor score was 1.6 point higher ([0.07–3.14], *p* = 0.04) in week 8 remitters compared to non-responders (Fig. 3A). One remitter was an outlier, and exclusion of this observation led to similar results (+1.76 point [0.19–3.32], *p* = 0.029). We did not find an association between baseline GAD-10 score and treatment response (regression coefficient 2.34 [−3.59 to 8.27], *p* = 0.43). We estimated a greater reduction in r∆HAMD_6_ of 18.3 points ([−37.3–0.79], *p* = 0.06) for anxious vs. non-anxious depression (Fig. 3B). Syndromal anxious depression at baseline was not associated with change in HAMD (−2.18 [−16.8–12.4], *p* = 0.77).

### Prediction of treatment outcome

AUC ranged between 0.64 and 0.67; inclusion of baseline anxiety measures resulted in an increase in AUC between 0 and 0.14. The best predictive value was found for non-responders versus all others when including baseline anxious depression as predictor; AUC with 0.79 [0.68–0.89] versus without 0.65 [0.49–0.81] anxious depression at baseline, although not significant *p* = 0.12 (not adjusted) (Table S1 and Fig. S2).

### Rescan

**Fig. 4:**
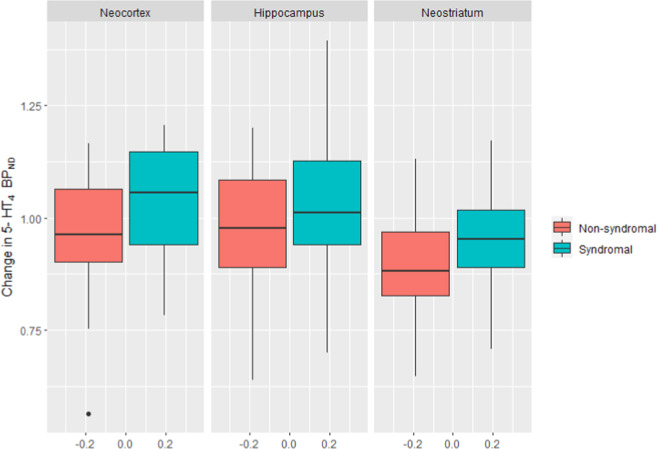
Change in 5-HT_4_R BP_ND_ in patients with/without syndromal anxious depression at baseline after 8 weeks of serotonergic antidepressant treatment. Regions of interest: neocortex, hippocampus and neostriatum. 1.00 on the *y*-axis represents no change in binding between baseline and week 8. Boxplot shows median bars and are not adjusted for covariates.

After 8 weeks of treatment, we found an association between the change in global BP_ND_ and syndromal anxious depression at baseline (*p* = 0.034). The distribution of the change in binding according to syndromal anxious depression is visualized in Fig. 4. In neocortex, patients with baseline syndromal anxious depression had a change in BP_ND_ of +3.5% vs. −4.7% in patients with non-syndromal depression, leading to a ratio of (1 + 3.5%)/(1–4.7%) = 8.5% (95%CI [−0.037 to 17.8], *p*.adj = 0.051) between groups. Correspondingly, in hippocampus there was a change in binding of +7.1% vs. −2.9%, and a ratio of 10.3%, (95%CI [−0.069 to 21.7], *p*.adj = 0.052), and for neostriatum −3.9% vs. −10.9, and a ratio of 7.8% (95%CI [0.04 to 16.1], *p*.adj = 0.048). One observation was an outlier and exclusion of this patient led to a slight decrease of the global effect from 8.51% to 5.54% (*p* = 0.068). We found no evidence for an association between change in BP_ND_ and anxious or non-anxious depression (*p* = 0.62), or any of the secondary outcomes (change in factor score (*p* = 0.46), change in GAD-10 score (*p* = 0.67)). In a post-hoc analysis, adjustment for duloxetine- or escitalopram treatment did not significantly influence the results.

## Discussion

In line with our hypothesis, a higher level of baseline concurrent anxiety symptoms was associated with lower 5-HT_4_R BP_ND_ across brain regions, both according to the self-rating questionnaire GAD-10 and the interview-based assessment of HAMD. The difference in BP_ND_ per one GAD-10 score was small (~0.5%), but a more clinically meaningful measure e.g. per ten GAD-10 score would generate a ~5% difference in BP_ND_, which is comparable to the magnitude of SSRI induced change seen in healthy men after 3 weeks of SSRI-intervention [22].

In line with rodent litterature [16–19], lower 5-HT_4_R agonistic capacity in humans may represent an endophenotype which is more prone to anxiety. We cannot, however, firmly establish whether low 5-HT_4_R binding is a trait or a state marker for anxiety in depression. Intriguingly, if 5-HT_4_R is an inverse proxy for cerebral 5-HT levels [22, 38], our observations suggest that high anxiety levels in depression are associated with higher-than-normal brain 5-HT levels and may correspond to the observation of serotonin-increase caused engagement of anxiety and fear-promoting circuit in the brain [48] and the well-known transiently increased anxiety following initiation of SSRI treatment. In line with our previous study [26], we propose that in the depressed or anxious state, higher brain 5-HT levels could reflect a compensatory mechanism to gain anxiolytic and antidepressant effects.

However, it is not possible in this study to establish whether the binding to 5-HT_4_R should be understood as an inverse proxy for 5-HT level and/or as a direct 5-HT_4_R effect.

We did not find that baseline anxiety was associated with worse treatment outcome; remitters had even a slightly higher baseline factor score than non-responders. The effect size, however, was small and the clinical importance of this observation is uncertain, especially since there was no difference in GAD-10 baseline score between non-responders and remitters (*p* = 0.43). Others have found that higher baseline anxiety score was associated with worse treatment response [49], although this observation might have been drug-specific since patients with higher baseline anxiety had better effect of escitalopram than another SSRI. In the STAR*D trial [8], patients with anxious depression were less likely to obtain remission. It is possible that the relatively young age (27, SD 8.2 years) of our patients, as age is known to increase the likelihood of a favourable outcome of SSRI’s [50–52], could influence this difference.

For the prediction analyses, we investigated the added value of concurrent anxiety at baseline to identify non-responders or remitters from all others but found no evidence for such a discriminative power. This may be due to the limited sample size. The largest added value was observed when using baseline anxious depression, with an improvement in AUC of 0.14, which is (while not statistically significant in our study) not negligible.

Even though we found no clear evidence for a difference in 5-HT_4_R binding between syndromal- and non-syndromal anxious depression at baseline (*p* = 0.11), patients with syndromal anxious depression had higher 5-HT_4_R binding after 8 weeks of treatment compared with their counterpart. Further, non-syndromal anxious patients had a decrease in 5-HT_4_R binding across all brain regions at rescan, while syndromal anxious patients showed lowered 5-HT_4_R binding in neostriatum only. This suggests that concurrent anxiety before drug treatment may influence the drug effect on the 5-HT_4_R.

Some limitations should be mentioned. First; without a control group, we cannot determine if anxiety score is negatively associated with 5-HT_4_R binding in general. Studies addressing this matter are needed. Second; the choice of treatment response criteria for non-responders and remitters can be debated. Our choice reduced the group sample size, potentially leading to a type-II error in the longitudinal- and rescan analyses. On the other hand, the rather restricted definitions allow for a more mechanistic interpretation of the results, where only the extreme response outcomes are included which was the á priori intention of this classification [28].

Third; the HAMD_17_ anxiety/somatization factor score has been criticized for not being stable across studies and to be weakly correlated to specific anxiety scales [53, 54]. Here, we included the factor score to allow for comparisons with other studies of anxious depression in addition to specific anxiety measures including GAD-10 score and syndromal anxious depression [34, 35] which provides diagnostic consistency across studies [31]. It is possible that other anxiety measures would have yielded different results, but we believe that this study covers a wide range of anxiety measures, both subjective and objective, which enhances the validity of the results. Fourth; results are not adjusted for analyses made for outcomes in other domains included in the main trial [28], and some *p*-values presented here were borderline and therefore with limited evidence. However, we only consider four outcomes (PET in three brain regions and concurrent anxiety). PET measurements were summarized using a latent variable, such that a single parameter was used to relate PET and anxiety with no need for adjustment at the global level. Fifth; for the rescan results, we cannot exclude the possibility that the effects occurred because of multiple factors contributing to 5-HT_4_R binding. It is expected that a change in binding may have several causes but even though we only assess associations here, anxiety seems to be a favourable candidate for future investigations.

In conclusion, we found that concurrent anxiety in patients with depression was negatively associated with cerebral 5-HT_4_R binding. We also found that remitting patients had higher baseline anxiety compared to those who did not respond to treatment. We did not find conclusive evidence for baseline anxiety as a useful predictor of treatment outcome in depression. At rescan, we found that concurrent syndromal anxious depression was associated with a global change in 5-HT_4_R binding after serotonergic antidepressant treatment. Overall, the 5-HT_4_R appears to be a promising neuroreceptor for further understanding the biological underpinnings of concurrent anxiety in patients with MDD.

## Supplementary information

Supplementary material

## Data Availability

All analyses were performed in R Studio (Version 1.4.1717). The code is available upon request.
