# Supplementary material for: Concurrent anxiety in patients with major depression and cerebral serotonin 4 receptor binding. A NeuroPharm-1 study
Source: Transl Psychiatry. 2022 Jul 11;12:273. doi: 10.1038/s41398-022-02034-5 (PMC9276803; doi:10.1038/s41398-022-02034-5)
Supplement: Supplementary file 1 — Supplementary material [file 41398_2022_2034_MOESM1_ESM.docx]

**Supplementary material**

**Table S1 and Figure S1-S2**

Table S1 shows area under the curve (AUC) for discriminating between treatment response, using a model with- or without baseline anxiety. Figure S1 is a CONSORT flow-diagram. Figure S2 shows ROC curves with and without anxiety as baseline predictor for treatment outcome (non-responders versus all others).

| **Groups** | **AUC [95%CI]** | **AUC [95%CI]** | **p(uncor)** |
| --- | --- | --- | --- |
|  | **With anxious depression** | **Without anxious depression** |  |
| **Non-responders vs. all others** | 0.79 [0.68-0.89] | 0.65 [0.49-0.81] | 0.12 |
| **Remitters vs. all others** | 0.66 [0.52-0.80] | 0.66 [0.51-0.80] | 0.66 |
|  | **With syndromal anxious depression** | **Without syndromal anxious depression** |  |
| **Non-responders vs. all others** | 0.66 [0.50-0.82] | 0.65 [0.49-0.80] | 0.92 |
| **Remitters vs. all others** | 0.66 [0.52-0.80] | 0.66 [0.51-0.80] | 0.64 |
|  | **With GAD-10 score** | **Without GAD-10 score** |  |
| **Non-responders vs. all others** | 0.67 [0.50-0.83] | 0.64 [0.49-0.81] | 0.43 |
| **Remitters vs. all others** | 0.67 [0.52-0.81] | 0.67 [0.51-0.80] | 0.91 |
|  | **With factor score** | **Without factor score** |  |
| **Non-responders vs. all others** | 0.69 [0.53-0.84] | 0.65 [0.50-0.81] | 0.74 |
| **Remitters vs. all others** | 0.70 [0.57-0.82] | 0.66 [0.51-0.80] | 0.31 |

**Table S1.** Area under the curve (AUC) for discriminating between treatment response using a model with or without baseline anxiety, i.e., either anxious depression, syndromal anxious depression, GAD-10 score and factor score. “All others” refers to the other two response-groups combined, e.g. remitters vs. all others (non-responders *and* intermediate responders). p-values are not adjusted for multiple comparisons.

**PATIENTS**

Screened for major depression, Aug 2016 - Jan 2019 (n= 259).

Enrollment

Excluded (n=159)

− Exclusion criteria (n=103)

− Declined (n=55)

− Other (n=1)

Included (n=100)

Allocation

Failed baseline examinations (n=8):

- excessive anxiety (n=2)

- unanticipated pregnancy (n=2)

- acute suicidal (n=1)

- radioligand failure (n=1)

- withdrawal (n=2)

**Baseline** **examinations**

Clinical examination, PET, MR (n=92*)

Anxiety measures:

- Factor score (n=91)

- GAD-10 questionnaire (n=89**)

- Syndromal/non-sydromal depression (42/49)

- Anxious/non-anxious depression (75/16)

Baseline

Dropout/Exclusion (n= 5):

- withdrawal of consent (n=1)

- spontaneous remission (n=1)

- acute suicidal (n=1)

- lost to follow-up (n=2)

Follow-Up

**Week 4 examinations**

Clinical examination (n=86)

36 early responders, 17 early non-responders, and 33 others.

Analyses

Duloxetine week 8:

Non-responder (n=5)

Intermediate responders (n=1)

Duloxetine rescan group:

Non-responder (n=2)

Intermediate responder (n=1)

*Excluded from baseline

- spontaneous remission (n=1)

** Missing data

- failed to fill out the questionnaire (n=2)

***Excluded from longitudinal analyses due to undetectable serum drug-levels (n=4)

- remitter (n=1)

- non-responder (n=1)

- intermediate responders (n=2)

****Excluded from rescan analyses (n=3):

- due to undetectable serum drug-levels: non-responder ^a^ (n=1) and intermediate responder ^b^ (n=1)

- scan failure: remitter (n=1)

Dropout/Exclusion (n= 4):

- withdrawal of consent (n=1)

- intolerable side-effects (n=1)

- lost to follow-up (n=2)

**Week 8 examinations**

Clinical examination (n=82)***

23 remitters, 14 non-responders, and 45 intermediate responders.

PET, MR (rescan, n=43)****

13 remitters, 6 non-responders, and 24 intermediate responders

Dropout/Exclusion (n=3)

- withdrawal of consent (n=2)

- acute psychosis (n=1)

**Week 12 examinations**

Last patient followed to April 2019

Clinical examination (n=75)

**Figure S1.** **CONSORT diagram**. ^a^ duloxetine, ^b^ escitalopram.


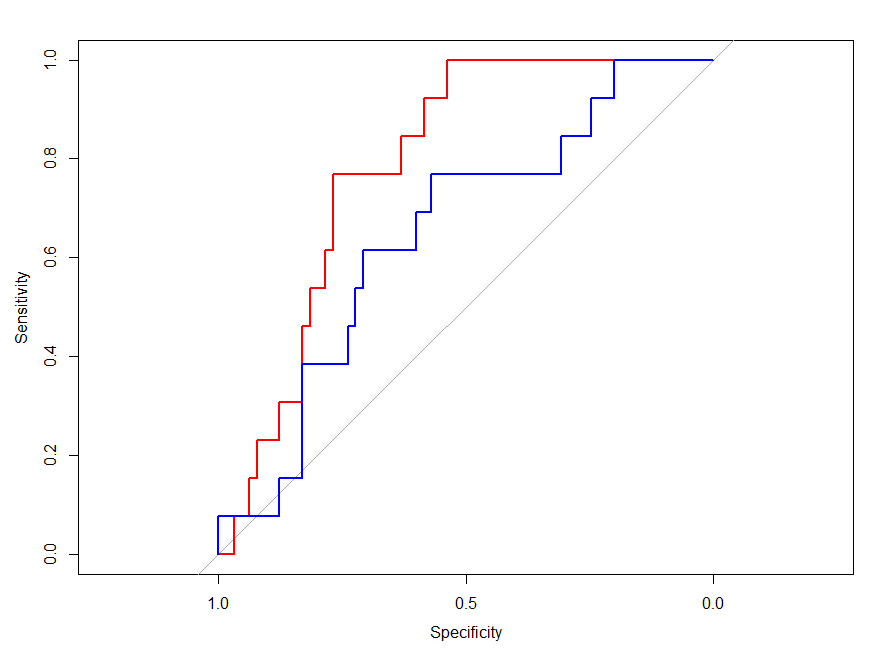


**Figure S2.** Receiver operating characteristic (ROC) curves with (red) and without (blue) anxiety as baseline predictor for treatment outcome (non-responders versus all others), where anxious depression at baseline showed a trend towards having discriminative power (p=0.12).
